# Excitation-Dependent Visible and Near-Infrared Luminescence of Dy^3+^/Pr^3+^ Co-Doped Borate Glasses

**DOI:** 10.3390/molecules31213972

**Published:** 2026-11-04

**Authors:** Markiyan Kushlyk, Bohdan Mahlovanyi, Yuriy Holovchak, Roman Golovchak, Ivanna Medvid, Dmytro Slobodzyan, Andriy Luchechko, Yaroslav Shpotyuk

**Affiliations:** 1 Department of Sensor and Semiconductor Electronics, Ivan Franko National University of Lviv, 107, Tarnavskoho St., 79017 Lviv, Ukraine; 2 Institute of Physics, University of Rzeszow, 1, Pigonia St., 35959 Rzeszow, Poland; 3 Department of Physics, Engineering and Astronomy, Austin Peay State University, Clarksville, TN 37044, USA

**Keywords:** borate glasses, dysprosium, praseodymium, photoluminescence, three-dimensional excitation-emission matrix, energy transfer, near-infrared emission

## Abstract

The optical properties of Dy^3+^- and Pr^3+^-doped (B_2_O_3_)_60_(ZnO)_10_(SrO)_10_(CaO)_10_(MgO)_10_ borate glasses were investigated using transmission, photoluminescence (PL), and three-dimensional excitation–emission matrix (EEM) spectroscopy. The transmission spectra revealed characteristic 4*f*–4*f* absorption bands of the rare-earth ions used. Dy^3+^-doped glasses exhibited prominent blue and yellow luminescence with the highest emission intensity observed for the 1.0 mol% Dy_2_O_3_ composition, which also exhibited near-white chromaticity. Pr^3+^-doped glasses showed weaker visible luminescence, with a dominant band in the orange region of the spectrum. In Dy^3+^/Pr^3+^ co-doped glasses, the visible Dy^3+^ emission was reduced, while the EEM maps revealed broadened excitation and additional near-infrared emission features.

## 1. Introduction

Rare-earth ion-doped glasses have attracted considerable attention for their distinctive optical and luminescent properties and wide range of applications in optoelectronic and photonic devices, including solid-state lasers, light-emitting diodes, and energy-efficient solutions [1,2,3,4,5,6,7]. Among various glass hosts, borate glasses are of particular interest because of their high transparency, relatively low melting points, good solubility for rare-earth ions, and high chemical stability [7,8]. The presence of BO_3_ and BO_4_ structural units in borate glasses leads to the formation of a complex three-dimensional glass network, which significantly influences the material’s physical and optical properties [9,10].

Alkaline earth oxides, such as SrO, CaO, and MgO, are widely utilized as network modifiers to tailor the structural and optical functionalities of borate glasses. The incorporation of strontium oxide (SrO) into the glass matrix is an effective strategy to alter the coordination state of boron structural units, thereby enhancing the material’s structural density, mechanical strength, and thermal stability [11]. Calcium oxide (CaO) plays a crucial role as a network modifier in borate-based glasses, optimizing their physicochemical performance and thermal stability through the controlled generation of non-bridging oxygens [12]. Among various network modifiers, magnesium oxide (MgO) has emerged as a highly effective component for inducing significant structural rearrangements in borate-based glass systems, thereby tuning their optical band gap energies [13]. The incorporation of modifiers such as ZnO can further improve glass stability, reduce non-radiative transitions, and enhance luminescence intensity [14].

In recent years, significant research attention has been devoted to developing materials for warm white light-emitting diodes (WLEDs), which offer advantages over conventional lighting sources due to their high brightness, high energy efficiency, long lifetimes, and environmental friendliness. However, traditional phosphor-based WLEDs often suffer from poor thermal stability and degradation of luminescent properties over time. Therefore, RE-doped glasses are considered promising alternatives owing to their high transparency, ease of fabrication, lack of halo effects, and stable luminescence characteristics [10,15,16]. The spectroscopic properties of Dy^3+^-doped glasses have been extensively investigated across various host matrices due to their strong potential for solid-state lighting, spectral conversion, and laser applications, as Dy^3+^ ions are among the most promising activators for white light generation, exhibiting intense emission bands in the blue (~480 nm) and yellow (~575 nm) spectral regions, corresponding to the ^4^F_9/2_ → ^6^H_15/2_ and ^4^F_9/2_ → ^6^H_13/2_ transitions, respectively [5,7,10,16]. At the same time, Pr^3+^ ions possess several metastable energy levels and exhibit emissions in the blue, green, orange, and red regions of the spectrum, depending on the matrix type. Owing to the proximity of certain energy levels of Dy^3+^ and Pr^3+^ ions, energy transfer between these ions may occur, enabling tunable emission colors and enhanced emission intensity [17]. In [15], lithium borate glasses co-doped with Dy^3+^/Pr^3+^ were reported to exhibit an intense emission band at 663 nm, attributed to energy transfer from Pr^3+^ to Dy^3+^, with the CIE chromaticity coordinates close to the white-light region. Similar results were reported for aluminoborate glasses [18], where warm-white emission and efficient non-radiative energy transfer mechanisms between Dy^3+^ and Pr^3+^ ions were observed. In addition, Pr^3+^-doped borate glasses demonstrate promising photoluminescent and photonic properties suitable for LED and laser applications [19]. However, these studies on oxide glasses focused mainly on visible emission, whereas infrared emission from Pr^3+^/Dy^3+^-doped systems has so far been reported primarily in low-phonon chalcogenide glasses [20]. In contrast, the present work uses three-dimensional (3D) excitation–emission matrix (EEM) spectroscopy to map the excitation–emission behavior over the UV–Vis–NIR range, revealing NIR emission at ~1000–1180 nm in the co-doped glass, which, to the best of our knowledge, has not been reported for Dy^3+^/Pr^3+^ co-doped borate glasses. The combination of these optical and luminescent properties makes such glasses promising for warm-white light sources and other optoelectronic devices operating in both the visible and NIR regions.

The (B_2_O_3_)_60_-(ZnO)_10_-(SrO)_10_-(CaO)_10_-(MgO)_10_ glass is a suitable host matrix for rare-earth ions due to its high optical transparency, structural versatility, and capacity to incorporate them. The incorporation of Dy^3+^ and Pr^3+^ ions provides an opportunity to tailor the glass’s emission characteristics over a broad spectral range and to investigate interactions between different luminescent centers. In particular, the combination of these ions enables the study of how their simultaneous incorporation affects excitation and emission processes, including potential interionic energy-transfer mechanisms. The melt-quenching method enables the preparation of homogeneous glasses with controlled dopant concentrations, providing a suitable basis for systematically studying the relationship between glass composition, rare-earth-ion concentration, energy-transfer processes, and luminescence efficiency. Therefore, this work aims to investigate the optical and luminescent properties of Dy^3+^-doped, Pr^3+^-doped, and Dy^3+^/ Pr^3+^ co-doped borate glasses and to elucidate the influence of co-doping on their visible and near-infrared (NIR) emission characteristics.

## 2. Results and Discussion

Chemical composition, dimensions, and physical properties (such as molar mass, density, and molar volume) of the studied glassy samples are presented in Table 1.

**Figure 1:**
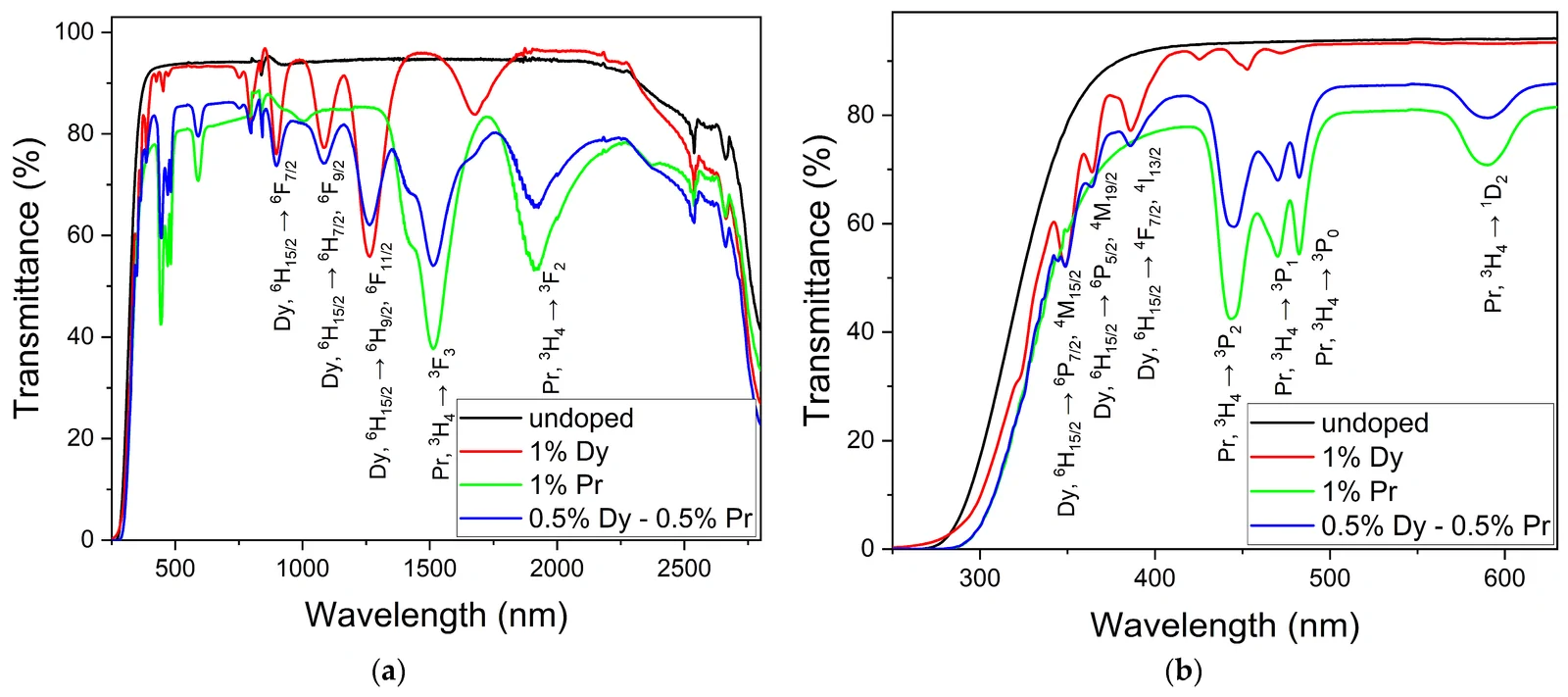
(**a**) Optical transmission spectra of B_59_:Dy_1_, B_59_:Pr_1_ single-doped and B_59_:Dy_0.5_/Pr_0.5_ co-doped borate glasses, (**b**) part of the transmission spectrum in the UV–Vis–NIR region.

The optical properties of the borate glasses doped with Dy^3+^ and/or Pr^3+^ ions were analyzed using UV–Vis–NIR transmission spectroscopy (Figure 1). These analyses confirmed the incorporation of both rare-earth ions into the glass matrix, as indicated by their characteristic absorption transitions in the spectra. The optical transmission spectra of the Dy^3+^-doped, Pr^3+^-doped, and Dy^3+^/Pr^3+^ co-doped glasses reveal distinct absorption features associated with the characteristic 4*f*–4*f* transitions of these rare-earth ions.

For Dy^3+^-doped glasses, absorption bands are observed in the range of 400–3200 nm, corresponding to transitions from the ^6^H_15/2_ ground state to various excited states, with the most intense peak occurring at 1265 nm, assigned to the ^6^H_15/2_ → (^6^H_9/2_, ^6^F_11/2_) transition. The presence of hypersensitive transitions, which follow established selection rules, provides insights into the local symmetry and bonding nature of the rare-earth ions in the glass matrix.

For Pr^3+^-doped glasses, the transmission spectra exhibit peaks at 446, 469, 482, 587, 1421, 1518, and 1919 nm, corresponding to transitions from the ^3^H_4_ ground state to higher-energy levels. The hypersensitive ^3^H_4_ → ^3^P_2_ transition in the violet-to-blue region shows a blue shift with increasing Pr^3+^ concentration due to the nephelauxetic effect, indicating a reduction in the covalent nature of the rare-earth–oxygen bond. It should be noted that the intense NIR absorption bands (^3^H_4_ → ^3^F_4_, ^3^F_3_, ^3^F_2_ transitions) are particularly relevant for optical applications [21].

In co-doped B_59_:Dy_0.5_/Pr_0.5_ glasses, the combined absorption features of both Dy^3+^ and Pr^3+^ ions result in a modified transmission profile, with overlapping absorption bands influencing the overall spectral response. Such overlap provides possible excitation channels for both ions, affecting luminescence properties. The variations in transmission spectra between single-doped and co-doped samples provide a foundation for optimizing the composition.

Previous studies on Dy^3+^-doped borate glasses have reported strong luminescence with distinct excitation bands that peak at 350 nm. Under this excitation, the emission spectra displayed characteristic bands at about 480 nm (blue), 580 nm (yellow), and a very weak band near 665 nm (red), corresponding to transitions in Dy^3+^ ions. The optimal Dy_2_O_3_ concentration for maximum luminescence was identified near 1.00 mol%, beyond which quenching effects reduced efficiency. The CIE 1931 chromaticity diagram analysis confirmed that Dy^3+^-doped glasses produced near-white light (slightly bluish tint), highlighting their suitability for WLED applications [7,10,22]. These results provide a reference for comparing the luminescence behavior of Pr^3+^ ions in similar glass matrices.

**Figure 2:**
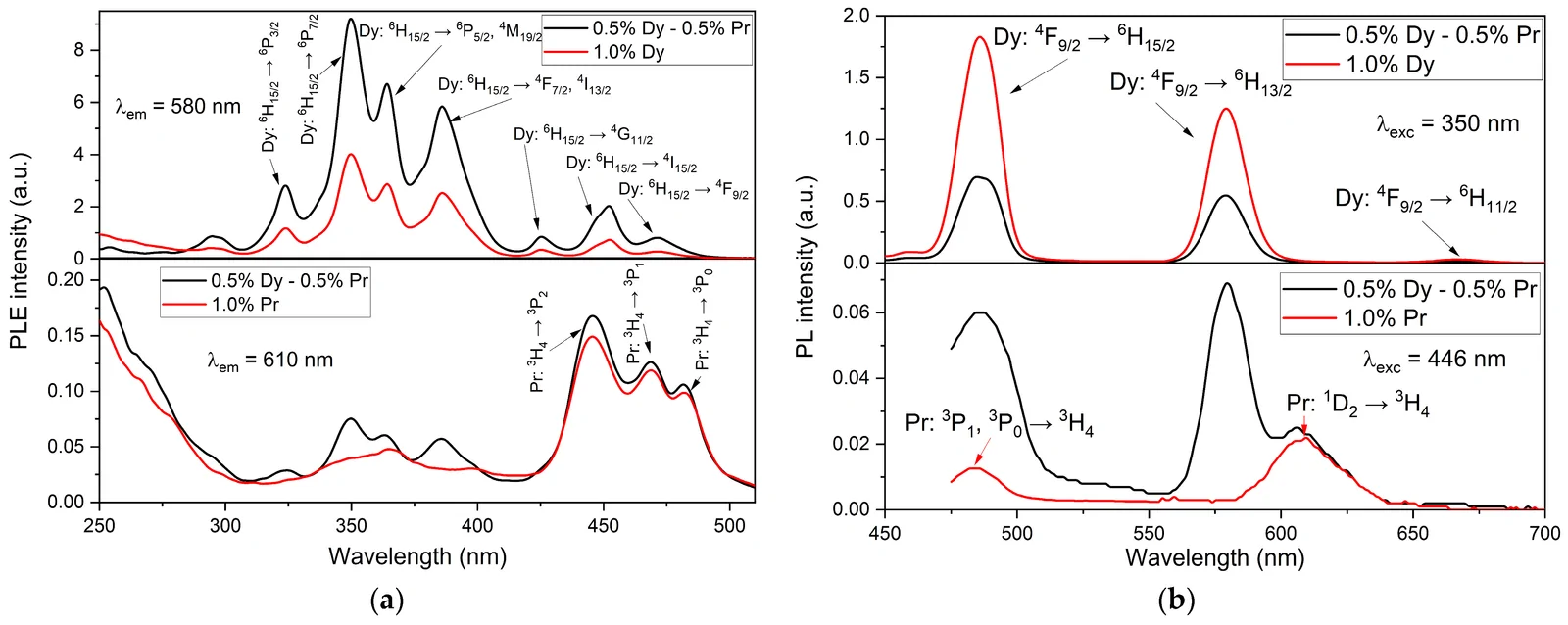
PL excitation (**a**) and emission (**b**) spectra of borate glasses doped with one type of Dy^3+^/Pr^3+^ ions and co-doped with Dy^3+^ and Pr^3+^ ions. The wavelengths λ_em_ = 580 nm and λ_exc_ = 350 nm correspond to Dy^3+^ ions, λ_em_ = 610 nm and λ_exc_ = 446 nm correspond to Pr^3+^ ions.

The PLE spectra (Figure 2a) reveal several characteristic excitation bands corresponding to intraconfigurational 4*f*–4*f* transitions of the rare-earth ions. The analysis of Pr^3+^-doped samples focused on their luminescence properties (Figure 2b) under 446 nm excitation. The emission spectra revealed a weak luminescence band centered at 610 nm, assigned to the ^1^D_2_ → ^3^H_4_ transition. Despite the presence of Pr^3+^, the overall luminescence intensity was relatively low. Samples co-doped with Dy^3+^ and Pr^3+^ exhibited similarly weak luminescence, likely due to energy transfer mechanisms between Dy^3+^ and Pr^3+^ ions. These mechanisms include NIR emission via the ^1^D_2_ → ^3^H_6_, ^1^D_2_ → ^3^F_3_, ^1^D_2_ → ^3^F_4_, and ^1^G_4_ → ^3^H_4_ transitions, thereby reducing the overall intensity of the visible emission [23].

The results show that Dy^3+^-doped borate glasses exhibit strong luminescence, with the highest emission intensity observed at a Dy_2_O_3_ concentration of 1.0 mol%. The strong luminescence is accompanied by near-white-light emission, making these glasses promising candidates for optical applications. Adding Pr^3+^ ions or co-doping with Dy^3+^ and Pr^3+^ results in weaker, partially orange luminescence, highlighting the distinct behavior of Pr dopants within the glass matrix.

**Figure 3:**
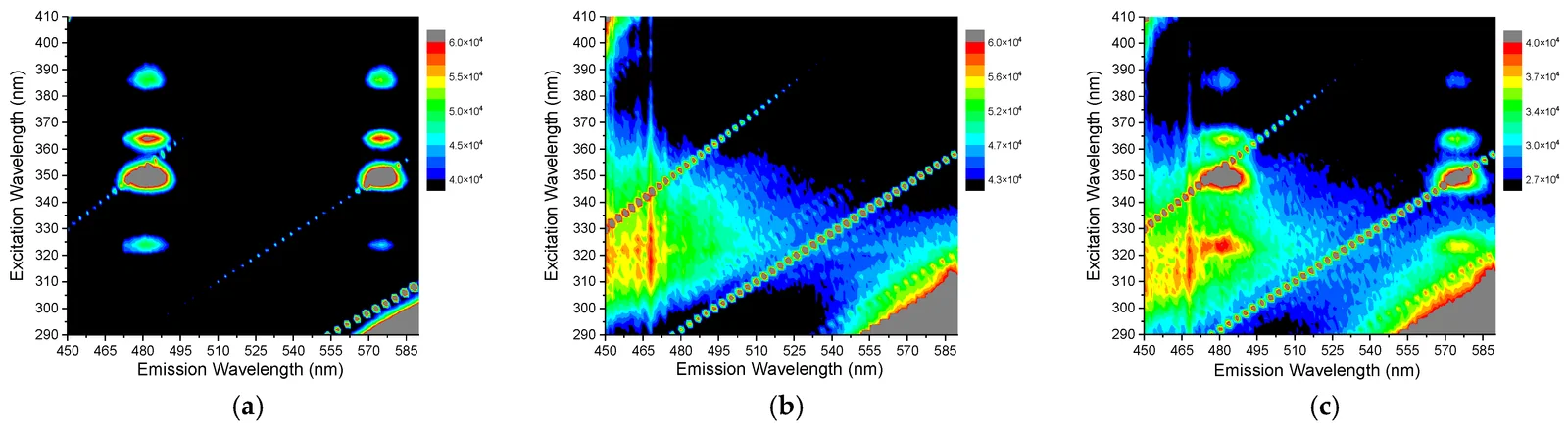
3D excitation–emission matrix photoluminescence maps of the studied borate glasses: (**a**) 1.0 mol% Dy_2_O_3_ single-doped glass; (**b**) 1.0 mol% Pr_2_O_3_ single-doped glass; (**c**) 0.5 mol% Dy_2_O_3_/0.5 mol% Pr_2_O_3_ co-doped glass.

To evaluate the optical response in the synthesized glasses, 3D EEM photoluminescence maps were recorded. The obtained 3D luminescent plots for the single-doped and co-doped glasses, in the UV–Vis spectral region, are presented in Figure 3. It should be noted that the 3D photoluminescence mapping of the undoped host matrix (B_2_O_3_)_60_-(ZnO)_10_-(SrO)_10_-(CaO)_10_-(MgO)_10_ demonstrates a completely dark profile in both the visible and NIR (up to 1600 nm) spectral regions. Apart from the intense diagonal track corresponding to the second-order diffraction artifacts (2λ_ex_ = λ_em_), no distinct intrinsic emission peaks or defect-related luminescence bands were detected under the applied excitation wavelengths. This confirms that the host borate matrix has high optical transparency and does not show emission transitions in the UV–Vis–NIR spectral range, making it an excellent host medium for rare-earth (RE^3+^) activators.

For the 1 mol% Dy_2_O_3_ single-doped sample, the EEM map reveals a set of sharp, discrete emission features characteristic of 4*f* → 4*f* intra-configurational transitions of Dy^3+^ ions. In the visible region (Figure 3a), two prominent emission bands are observed: a blue emission band centered at *λ_em_* ≈ 480 nm, attributed to the magnetically-allowed ^4^F_9/2_ → ^6^H_15/2_ transition and an intense yellow emission band located at *λ*_em_ ≈ 575 nm, originating from the hypersensitive forced electric-dipole ^4^F_9/2_ → ^6^H_13/2_ transition (∆*L* = 2, ∆*J* = 2) [24]. The excitation EEM profile shows that the ^4^F_9/2_ metastable state is most effectively populated via absorption bands in the near-UV and violet regions, including features near *λ*_ex_ ≈ 325, 350, 365, and 385 nm, with the strongest maximum around 350 nm, corresponding to the ^6^H_15/2_ → ^6^P_7/2_/^4^M_19/2_ transition.

The EEM map of the 1 mol% Pr_2_O_3_ single-doped sample shows a fundamentally different topology, characterized by broadband emission features (Figure 3b). It demonstrates a broad emission spanning from 450 nm to the red region (~600 nm) with a fine structure in the 440–480 nm range, governed by radiative transitions from the ^3^P_0_ (and partially ^1^D_2_) states of Pr^3+^ to the lower ^3^H_J_ and ^3^F_J_ levels. The excitation map reveals that these transitions are strongly driven by a broad absorption band in the blue spectral region (440–480 nm), corresponding to the ground state absorption ^3^H_4_ → ^3^P_J_. Additionally, an excitation channel emerges in the deep-UV region (λ_ex_ < 340 nm), which may be associated with interconfigurational 4f → 5d transitions or host-to-activator energy transfer.

For the co-doped glass composition containing 0.5 mol.% Dy_2_O_3_ and 0.5 mol.% Pr_2_O_3_, the 3D luminescent topography reflects a superposition of both activator networks. In the visible range, the distinct “spot-like” sharp emission peaks of Dy^3+^ (such as 480 nm and 575 nm peaks) become superimposed on the broader emission background of Pr^3+^. Due to the bidirectional decrease in individual activator concentrations (from 1% to 0.5%), the overall absolute visible luminescence intensity decreases (Figure 3c).

**Figure 4:**
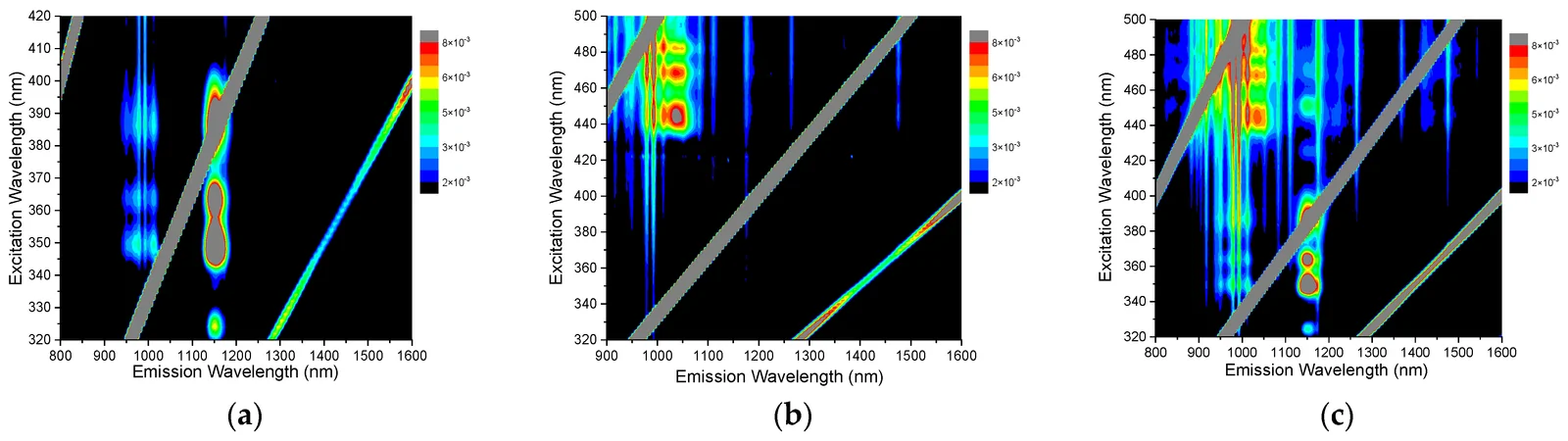
3D excitation–emission matrix photoluminescence maps of the borate glass samples in the NIR spectrum: (**a**) 1.0 mol% Dy_2_O_3_ single-doped glass; (**b**) 1.0 mol% Pr_2_O_3_ single-doped glass; (**c**) 0.5 mol% Dy_2_O_3_ / 0.5 mol% Pr_2_O_3_ co-doped glass.

In the NIR spectrum, additional radiative relaxation pathways are observed in Dy-single-doped samples, which are characteristic of the ^4^F_9/2_ energy level. Specifically, distinct emission bands appear around 980 nm and 1010 nm (corresponding to ^4^F_9/2_ → ^6^F_7/2_ and ^4^I_15/2_ → ^6^F_5/2_ transitions, respectively). Furthermore, a strong, well-defined NIR luminescence is observed in the 1140–1180 nm range, extending the functional applications of the Dy^3+^-doped system into the NIR photonics (Figure 4a).

In Dy^3+^-doped borate glasses, the NIR luminescence observed in the 1140–1180 nm range can be assigned primarily to the Dy^3+ 4^F_9/2_ → ^6^F_5/2_ transition. The high phonon energy of the borate host efficiently drives non-radiative multiphonon relaxation from upper excited states to successfully populate this ^4^F_9/2_ emitting level. Concurrently, the highly asymmetric local environment of the borate glass network relaxes parity selection rules, resulting in the characteristic inhomogeneous broadening of this specific emission profile.

The NIR EEM map (Figure 4b) reveals strong luminescence features with distinct emission lines around 980 nm and 1020 nm, which correspond to the characteristic transitions of Pr^3+^ ions and are effectively populated over a broad UV-to-green excitation range (260–500 nm). Literature on Pr^3+^-doped glasses reports nearby ^1^D_2_ → ^3^F_3_ and ^1^D_2_ → ^3^F_4_ emissions and possible overlap with ^1^G_4_ → ^3^H_4_ near ~1.0–1.05 µm [4].

The NIR map of the co-doped glass shows combined behavior (Figure 4c). The spectral structure reveals an elongated excitation profile along the *λ_ex_* axis, extending from approximately 320 nm to 480 nm, which is associated with infrared emission bands at *λ_em_* ≈ 1000–1050 nm and *λ_em_* ≈ 1160 nm. This broad excitation response indicates that both Dy^3+^ and Pr^3+^ absorption channels contribute to the observed NIR emission. Consequently, the observed broadband NIR emission under UV/visible excitation indicates the potential of the co-doped system for spectral conversion and NIR photonic applications.

The spectral map reveals that the dominant luminescent feature in this regime is localized within the 980–1020 nm wavelength band. This primary emission achieves its maximum PL intensity when pumped at specific excitation channels, notably around 440–450 nm and 475–495 nm. A secondary, lower-intensity emission band is presented in the 1160–1180 nm range. Within the analyzed visible excitation window, this transition—primarily ascribed to the Dy^3+ 4^F_9/2_ → ^6^F_5/2_ decay channel—exhibits a localized excitation maximum at *λ_ex_* ~ 430–440 nm. Weak emission is also observed at approximately 1470–1480 nm under excitation in the 420–500 nm range, accompanied by trace background emission faintly discernible near 1320–1350 nm under the same excitation conditions (Figure 4c).

The two primary excitation bands observed in the blue region (440–450 nm and 475–490 nm) originate from the direct, intraconfigurational 4*f*–4*f* ground-state absorption transitions of both ions. Because both rare-earth ions possess excited energy levels that strongly absorb blue light, the broad features seen in the excitation–emission PL map are the result of the superposition of multiple overlapping transitions from both dopants. Furthermore, the amorphous nature of the multicomponent BZnSrCaMg glass host induces inhomogeneous broadening, which merges these adjacent Stark-split transitions into continuous bands.

The 430–450 nm excitation band can be regarded as a convolution of two bands: Pr^3+^ (the ^3^H_4_ → ^3^P_2_ transition) and Dy^3+^ (the ^6^H_15/2_ → ^4^I_15/2_ transition). The first one is typically among the strongest absorption channels for praseodymium in the visible spectrum, peaking around 442–445 nm. The absorption peak for dysprosium typically manifests around 450–452 nm. Their overlap limits ion-specific attribution of the corresponding EEM excitation band.

The 475–490 nm lower-energy excitation band is dominated by the strong absorption states of praseodymium, with a secondary overlapping tail from dysprosium. This region encompasses the characteristic Pr^3+ 3^H_4_ → ^3^P_0_ transition (which strongly peaks around 482 nm), as well as the closely adjacent ^3^H_4_ → ^3^P_1_ and ^3^H_4_ → ^1^I_6_ transitions (located around 468–475 nm) [25]. The ^6^H_15/2_ → ^4^F_9/2_ transition of Dy^3+^ ions typically peaks slightly lower (around 470–475 nm), and its lower-energy tail significantly overlaps with the Pr absorption profile (^3^H_4_ → ^3^P_0_).

Because both Pr^3+^ and Dy^3+^ are simultaneously excited by these wavelengths in the blue region, both ions are promoted to their respective excited states. Such spectral overlap may favor interionic energy transfer and cross-relaxation between Pr^3+^ and Dy^3+^ ions, thereby contributing to the NIR emission observed in the co-doped glass.

**Figure 5:**
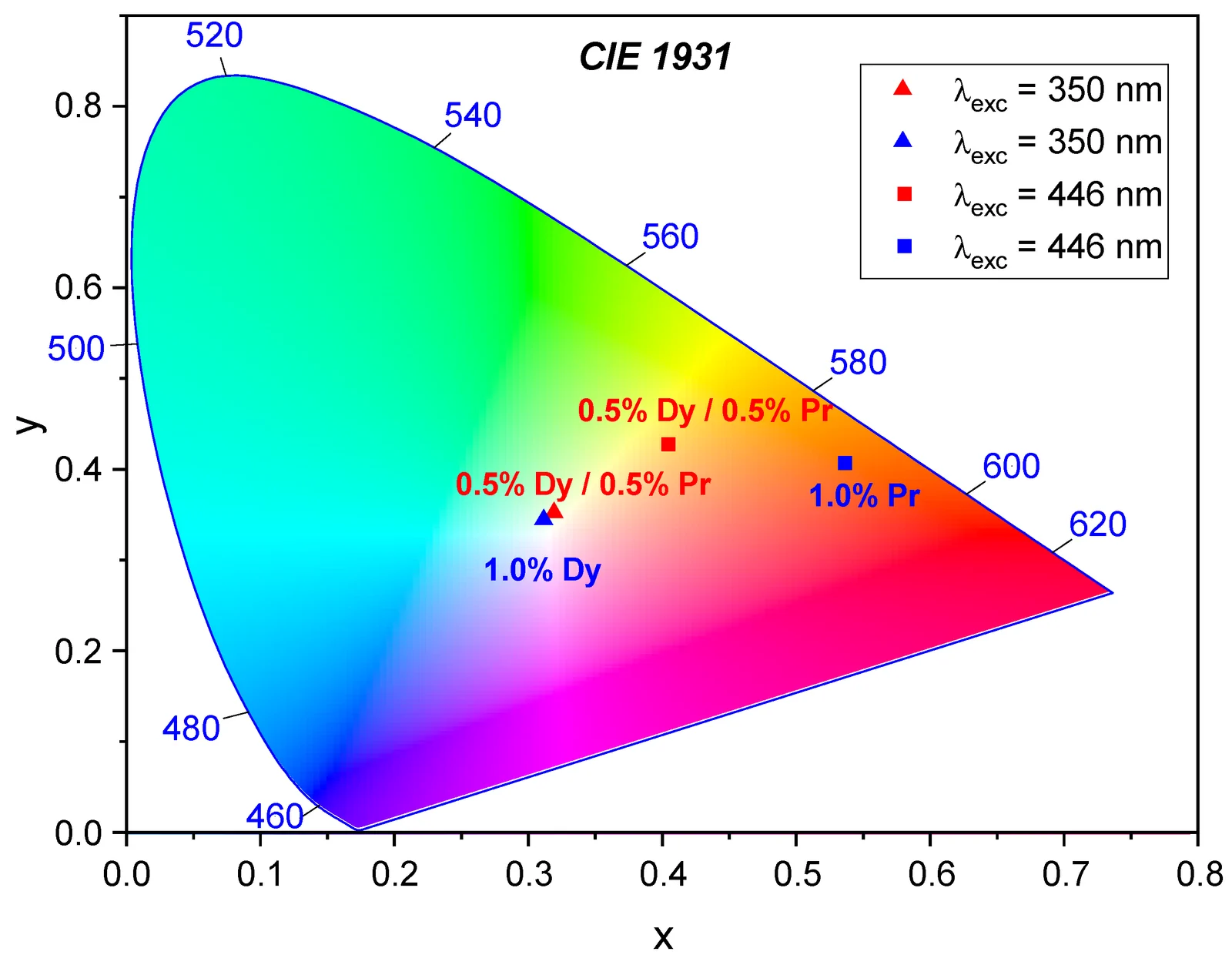
The CIE 1931 chromaticity diagram of Dy^3+^- and Pr^3+^-doped borate glasses obtained under different concentrations of dopants and excitation wavelength.

The CIE 1931 chromaticity diagram illustrates the emission color coordinates of Dy^3+^- and Pr^3+^-doped borate glasses measured under different excitation wavelengths (Figure 5). Under 350 nm excitation (triangles), both samples exhibit chromaticity coordinates close to the central region of the CIE diagram, near the white-light reference point (x, y) ≈ (0.33, 0.33). This indicates near-white chromaticity, with small differences between the Dy^3+^-doped and Dy^3+^/Pr^3+^ co-doped glasses. In contrast, under 446 nm excitation (squares), the Dy^3+^/Pr^3+^ co-doped glass exhibits chromaticity in the yellow-orange region, while the Pr^3+^-doped glass shows chromaticity in the orange region.

The chromaticity diagram (Figure 5) demonstrates that the emission color of the investigated borate glasses can be modified by varying the Dy^3+^/Pr^3+^ dopant composition and excitation wavelength, indicating that the emission color can be tuned by appropriately selecting the excitation conditions and glass composition. These results highlight the potential of Dy^3+^- and Pr^3+^-doped borate glasses as color-tunable luminescent materials for phosphor and optoelectronic applications.

**Figure 6:**
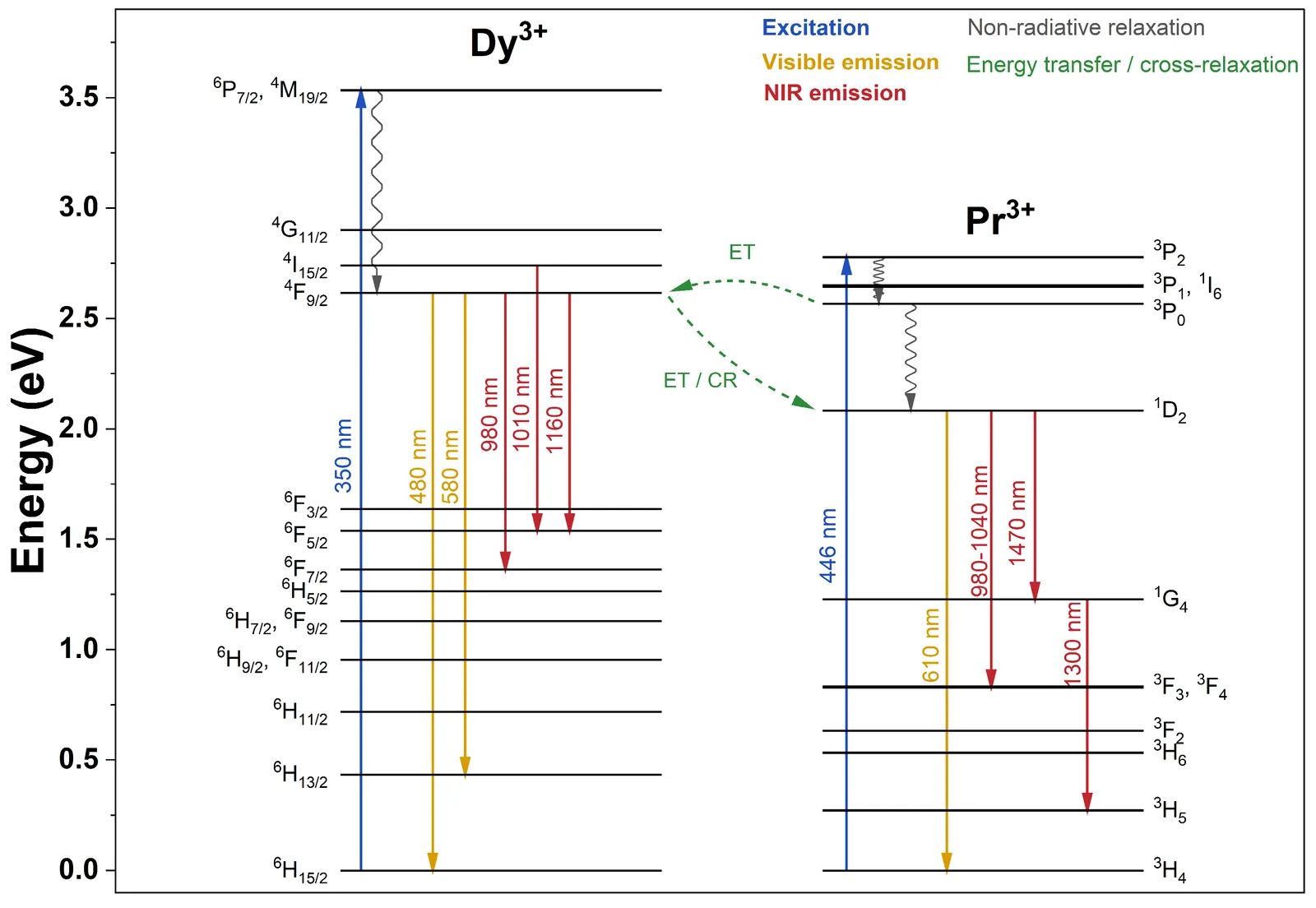
Energy levels diagram of Dy^3+^ and Pr^3+^ ions (excitation pathways—blue arrows, emission transitions—orange and red arrows, non-radiative relaxation processes—wavy gray arrows, and possible interionic energy-transfer mechanisms—green dashed arrows.

The diagram in Figure 6 illustrates the electronic energy levels of trivalent dysprosium (Dy^3+^) and praseodymium (Pr^3+^) ions, along with their excitation and emission pathways, non-radiative relaxation processes, and possible interionic energy-transfer mechanisms. Horizontal lines denote the electronic multiplet levels, solid vertical arrows denote spectrally determined excitation and emission bands, wavy arrows represent plausible nonradiative relaxation, while dashed interionic arrows are only proposed pathways of relaxation and energy-transfer processes, based on the analysis of excitation and emission spectra in the UV–visible–NIR spectral range.

It should be noted that the dashed interionic arrows in Figure 6 are energy transfer hypotheses based on spectral overlap of bands and approximate ion energy-level matching. The steady-state excitation and emission spectra obtained in this work do not directly establish the energy-transfer/relaxation direction. Confirmation of a proposed mechanism would require a concentration series of samples and time-resolved decay measurements to determine lifetimes and transfer efficiencies.

The left-hand side shows the energy-level scheme of Dy^3+^. Its ground state is ^6^H_15/2_, and the excited states include the ^6^H_J_, ^6^F_J_, ^4^F_9/2_, ^4^I_15/2_, ^4^G_11/2_, and higher-lying multiplets. Excitation at 350 nm (blue arrow): promotes Dy^3+^ ions from the ground state to a high-energy excited state, labeled approximately ^6^P_7/2_ and/or ^4^M_19/2_ in the diagram. Excited ions non-radiatively relax to lower-lying states through phonon-assisted processes, potentially populating the metastable ^4^F_9/2_ level. The visible emissions around 480 and 575 nm (orange arrows) and NIR emission around 980, 1010, and 1160 nm (red arrows) are characteristic Dy^3+^ transitions.

The right-hand side presents the energy-level scheme of Pr^3+^. The principal multiplets include the ground state ^3^H_4_, the ^3^H_5_, ^3^H_6_, ^3^F_J_, ^1^G_4_, ^1^D_2_, and higher-lying ^3^P_J_ states. Excitation at 446 nm (blue arrow) excites Pr^3+^ ions from the ground state to the ^3^P_J_ manifold, principally the ^3^P_2_ region. Non-radiative relaxation (wavy gray arrows) transfers population from the higher excited states toward lower levels, including ^1^D_2_. It should be noted that the ^1^D_2_ state is important because it contributes to both visible (orange arrows, emission at 610 nm associated with the ^1^D_2_ → ^3^H_4_) transitions) and NIR (red arrows, 980–1040 nm emission, associated with transitions from ^1^D_2_ to the ^3^F_J_ manifold) emission. The emissions at about 1300 nm and 1470 nm are assigned to the ^1^G_4_ → ^3^H_5_ and ^1^D_2_ → ^1^G_4_ transitions, respectively, involving lower-lying Pr^3+^ multiplets.

The green dashed arrows represent possible interionic energy transfer (ET) and cross-relaxation (CR) pathways between Dy^3+^ and Pr^3+^. The energy-level diagram illustrates possible interaction between Dy^3+^ and Pr^3+^ ions through resonant energy transfer (ET), which could redistribute excitation energy and modify their relative emission intensities (Figure 3, Figure 4 and Figure 5). The process is governed by the energy matching of electronic transitions, spectral overlap, and the distance between neighboring ions.

In particular, Dy^3+^ → Pr^3+^ energy transfer may occur from the Dy^3+ 4^F_9/2_ level to neighboring Pr^3+^ ions, promoting them to the ^3^P_J_ manifold, as suggested by the spectral overlap between the Dy^3+^ emission near 480 nm and the Pr^3+^ absorption bands in the 440–490 nm region. The reverse Pr^3+^ (^3^P_0_) → Dy^3+^ (^4^F_9/2_) transfer is also energetically possible. Such bidirectional interaction could account for the broadened excitation response of the co-doped glass. This makes Dy^3+^/Pr^3+^ co-doped glasses interesting for excitation-tunable visible and NIR emission.

## 3. Materials and Methods

### 3.1. Glass Synthesis

The undoped (B_2_O_3_)_60_-(ZnO)_10_-(SrO)_10_-(CaO)_10_-(MgO)_10_ and RE-doped (B_2_O_3_)_59_-(ZnO)_10_-(SrO)_10_-(CaO)_10_-(MgO)_10_ glass compositions were synthesized using the melt-quenching technique, ensuring the homogeneity and structural integrity of the prepared materials. The original borate glass matrix was single-doped with (Dy_2_O_3_)-1.0 mol%, (Pr_2_O_3_)-1.0 mol%, and co-doped with (Dy_2_O_3_)-0.5 mol% and (Pr_2_O_3_)-0.5 mol%. (Table 1).

**Figure 7:**
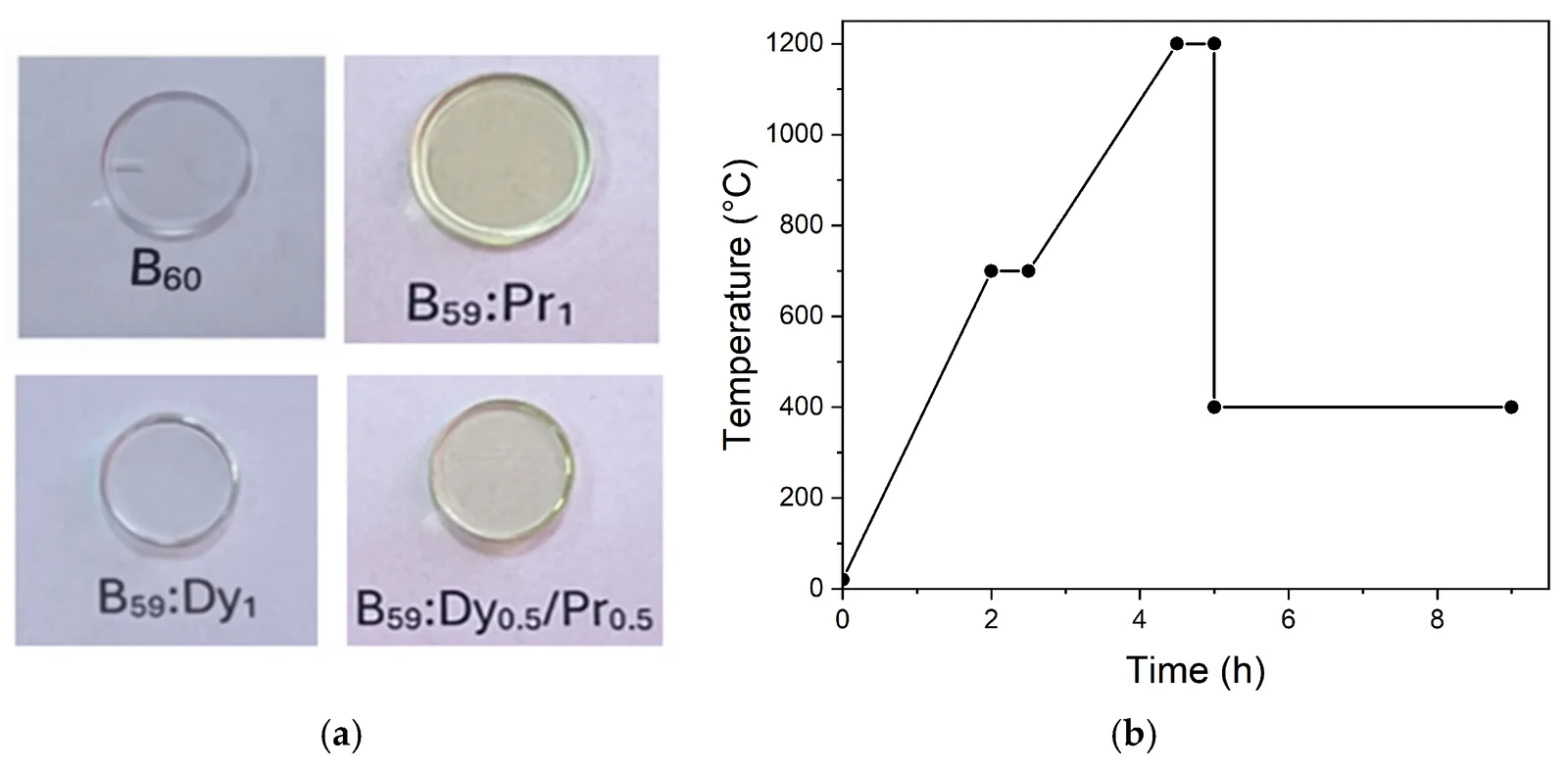
Photography of synthesized glass samples (**a**) and temperature map of glass synthesis (**b**).

The synthesis procedure involved weighing high-purity raw materials in precise stoichiometric proportions, including B_2_O_3_, ZnO, SrO, CaO, MgO, and the respective rare-earth oxides. The powdered mixture was melted and homogenized in Al_2_O_3_ crucibles in a high-temperature furnace. The thermal treatment process (Figure 7b) included an initial 2-h heating phase from room temperature to approximately 700 °C, followed by a second heating phase to approximately 1200 °C, with a 1-h dwell to ensure successful completion of melting and homogenization. The molten glass was then rapidly quenched into carbon molds to prevent crystallization, followed by a 4-h annealing phase at approximately 400 °C to relieve internal stresses.

After cooling, the glass samples were polished to achieve high-quality optical surfaces (Figure 7a), enabling precise optical and luminescent measurements in subsequent characterization studies. The photograph of the samples (Figure 7a) was taken with a smartphone camera under ambient daylight using automatic settings and is intended for illustrative purposes only.

### 3.2. Characterization Techniques

Sample thicknesses required for absorption coefficient calculations were measured with a digital micrometer (±0.01 mm accuracy) and averaged across multiple readings. Molar mass was determined using stoichiometric calculations of the oxide components, while density was assessed using Archimedes’ principle by comparing weights in air and deionized water. Molar volume was derived from the molar mass-to-density ratio, providing insights into the glass matrix structure.

**Figure 8:**
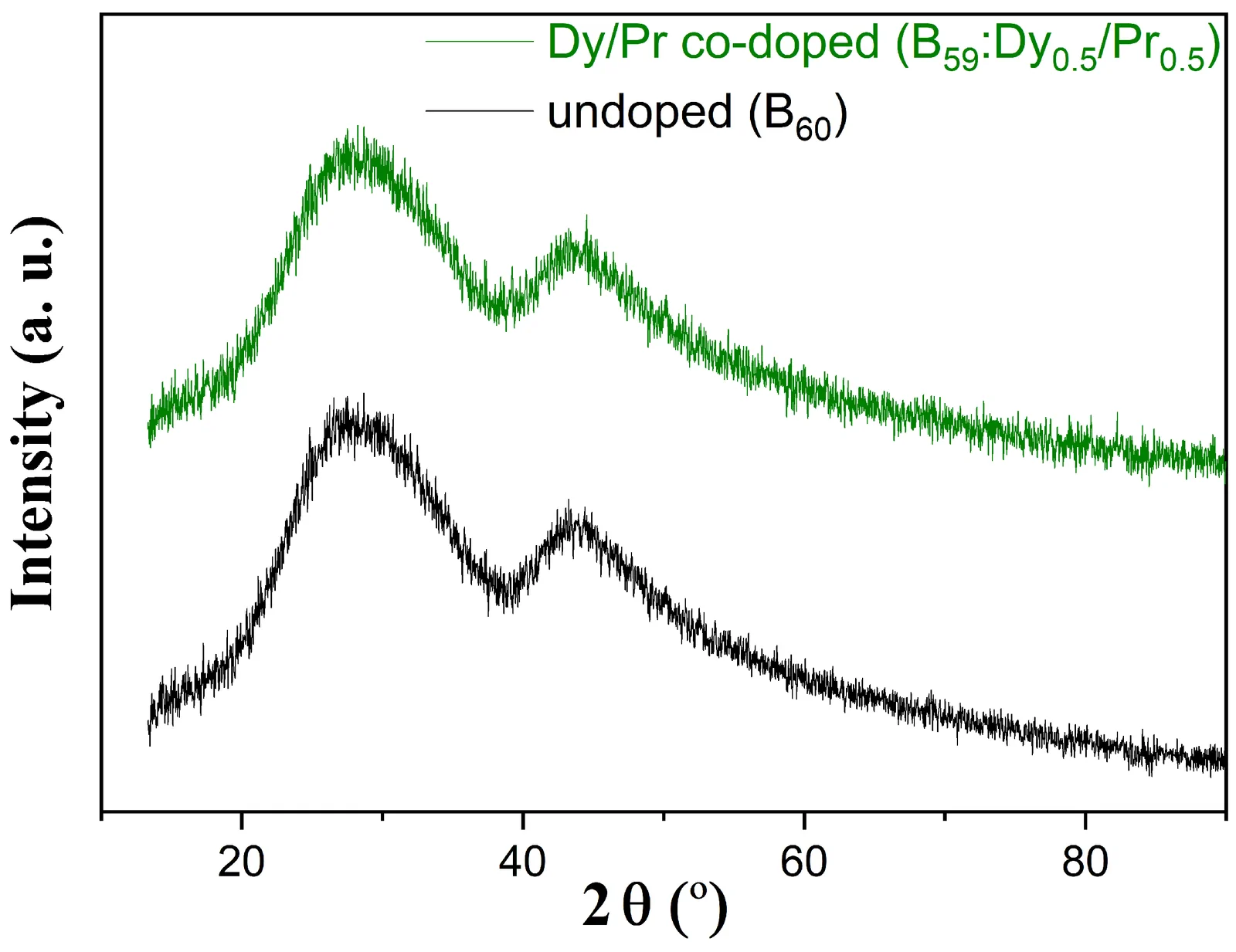
The X-ray diffraction pattern of the studied B_60_ (undoped) and B_59_:Dy_0.5_/Pr_0.5_ (Dy/Pr co-doped) samples.

The amorphous nature of the synthesized glasses was verified by X-ray diffraction using a Bruker D8 Advance (Bruker AXS GmbH, Karlsruhe, Germany) with Cu Kα radiation (λ = 1.5406 Å) in the 2θ range of 10–90° 2θ. The XRD patterns of the undoped B_60_ and Dy/Pr co-doped B_59_:Dy_0.5_/Pr_0.5_ glasses are shown in Figure 8.

The patterns of undoped and Dy/Pr co-doped samples exhibit two broad, diffuse halos centered at approximately 28° and 44° (2θ) and no sharp Bragg reflections, confirming the non-crystalline character of the host matrix and the disordered atomic arrangement typical of glassy systems. The XRD pattern of the co-doped glass confirms that the incorporation of rare-earth oxides does not induce crystallization. An amorphous structure of the investigated samples is further supported by their visual transparency and homogeneity (Figure 7a).

The optical properties of Dy^3+^- and Pr^3+^-doped borate glasses were investigated using transmission and photoluminescence (PL) spectroscopy. Optical transmission spectra (200–3200 nm, 1 nm resolution) were recorded using an Agilent Cary-5000 spectrometer (Agilent Technologies, Inc., Santa Clara, CA, USA). Photoluminescence excitation (PLE) and photoluminescence (PL) spectra (220–820 nm) were recorded using a CM2203 spectrofluorometer (SOLAR, Minsk, Belarus), with a 150 W xenon lamp as the excitation source and a Hamamatsu R928 photomultiplier (Hamamatsu Photonics K.K., Hamamatsu City, Shizuoka, Japan) for detection. Excitation–emission matrix (EEM) spectroscopy was performed using a Horiba Fluorolog-3 spectrofluorometer (HORIBA Jobin Yvon S.A.S., Longjumeau, France) equipped with a xenon short-arc lamp as the light source. For the NIR emission measurements (800–1600 nm), the liquid nitrogen-cooled Jobin Yvon InGaAs DSS-IGA020L NIR detector (HORIBA France SAS, Palaiseau, France) was used. The emission data were further analyzed to generate the CIE chromaticity diagram, providing a visual representation of color coordinates and assessing the glass’s potential for white-light emission applications. Therefore, the characterization stage focused on analyzing borate glasses using optical spectrophotometry, transmission measurements, and photoluminescence spectroscopy to evaluate their optical and luminescent properties. The aim of this approach was to identify and select samples with the most favorable optical-luminescence characteristics for potential applications.

## 4. Conclusions

In this study, multi-component (B_2_O_3_)_60_(ZnO)_10_(SrO)_10_(CaO)_10_(MgO)_10_ borate glasses, single-doped and co-doped with Dy^3+^ and Pr^3+^ ions, were successfully synthesized via the conventional melt-quenching technique. UV–Vis–NIR transmission spectroscopy revealed characteristic 4*f*–4*f* absorption transitions of Dy^3+^ and Pr^3+^ ions, demonstrating effective incorporation of rare-earth activators into the borate host network.

The photoluminescence studies show that Dy^3+^-doped glasses exhibit prominent blue (~480 nm) and yellow (~575 nm) emissions under near-UV excitation, making them highly promising candidates for near-white-light generation in WLED applications. Conversely, samples doped solely with Pr^3+^ ions exhibited a dominant orange-red emission near 610 nm, with relatively low intensity.

Three-dimensional excitation–emission matrix (EEM) mapping provided a comprehensive characterization of the excitation-dependent luminescence of Dy^3+^- and Pr^3+^-doped, and Dy^3+^/Pr^3+^-doped, borate glasses across the visible and near-infrared spectral regions. This approach enabled the identification of characteristic emission bands, their optimal excitation wavelengths, and overlapping excitation–emission features that are difficult to distinguish using conventional photoluminescence spectroscopy. In particular, EEM analysis revealed distinct NIR emission bands in the 1000–1180 nm range and a broadened excitation response in the co-doped glass, demonstrating the potential for excitation-dependent spectral tuning. The observed redistribution of visible and NIR emission features suggests possible interactions between Dy^3+^ and Pr^3+^ luminescent centers, although the specific energy-transfer mechanisms require further experimental verification. These findings highlight the value of 3D EEM spectroscopy for understanding the complex luminescence behavior of rare-earth-doped glasses and identifying promising compositions for visible–NIR photonic applications.

Consequently, while Dy^3+^-doped compositions are optimal for solid-state lighting (WLEDs), Dy^3+^/Pr^3+^-co-doped borate glass systems are promising candidates for spectral conversion and NIR photonic applications.

## Figures and Tables

**Table 1:** Chemical composition and physical properties of the studied glasses.

Sample ID	Composition	Thickness, mm	Molar Mass, g/mol	Density, g/cm^3^	Molar Volume, cm^3^/mol
B_60_	(B_2_O_3_)_60_-(ZnO)_10_-(SrO)_10_-(CaO)_10_-(MgO)_10_	1.20	69.899	2.728	25.62
B_59_:Dy_1_	(B_2_O_3_)_59_-(ZnO)_10_-(SrO)_10_-(CaO)_10_-(MgO)_10_-(Dy_2_O_3_)_1_	1.76	72.933	2.780	26.23
B_59_:Pr_1_	(B_2_O_3_)_59_-(ZnO)_10_-(SrO)_10_-(CaO)_10_-(MgO)_10_-(Pr_2_O_3_)_1_	1.88	72.501	2.769	26.18
B_59_:Dy_0.5_/Pr_0.5_	(B_2_O_3_)_59_-(ZnO)_10_-(SrO)_10_-(CaO)_10_-(MgO)_10_-(Dy_2_O_3_)_0.5_-(Pr_2_O_3_)_0.5_	2.00	72.717	2.792	26.05

## Data Availability

The original contributions presented in this study are included in the article. Further inquiries can be directed to the corresponding author.

## Abbreviations

The following abbreviations are used in this manuscript:

RE	Rare-earth
WLEDs	White light-emitting diodes
PL	Photoluminescence
PLE	Photoluminescence excitation
EEM	Excitation emission matrix
NIR	Near infrared
